# Healthy aging determinants and disability among older adults: SABE Colombia

**DOI:** 10.26633/RPSP.2021.98

**Published:** 2021-09-01

**Authors:** Fernando Gómez, David Osorio-García, Luisa Panesso, Carmen-Lucia Curcio

**Affiliations:** 1 Universidad de Caldas Manizales Colombia Universidad de Caldas, Manizales, Colombia

**Keywords:** Healthy aging, social determinants of health, disabled persons, Latin America, Colombia, Envejecimiento saludable, determinantes sociales de la salud, personas con discapacidad, América Latina, Colombia, Envelhecimento saudável, determinantes sociais da saúde, pessoas com deficiência, América Latina, Colômbia

## Abstract

**Objective.:**

To identify the main factors associated with disability in older adults in Colombia, adjusted according to structural and intermediary determinants of healthy aging.

**Methods.:**

This study used cross-sectional data from 23 694 adults over 60 years of age in the SABE Colombia nationwide survey. Structural determinants such as demographic and socioeconomic position variables were analyzed. Intermediary variables were classified into three blocks: intrinsic capacity, physical and built environment, and health care systems. Data analysis employed multivariate logistic regression.

**Results.:**

The prevalence of overall disability was 21% for activities of daily living, 38% for instrumental activities of daily living, and 33% for mobility disability. Disability was associated with sociodemographic structural determinants such as older age, female sex, rural residence, never married/divorced, living alone, low educational level, and Indigenous/Black ethnicity. With regard to determinants of socioeconomic position, net low income, poor socioeconomic stratum, insufficient income perception, and a subsidized health insurance scheme exerted a major influence on disability. Intermediary determinants of intrinsic capacity, such as poor self-rated health, multimorbidity, low grip strength, sedentary lifestyle, early childhood economic adversity, no social support, and no participation in activities, were significantly associated with disability.

**Conclusions.:**

Actions that affect the main factors associated with disability, such as reducing health inequities through policies, strategies, and activities, can contribute significantly to the well-being and quality of life of Colombian older adults.

## RESUMEN

**Objetivo.:**

Determinar los principales factores asociados con la discapacidad en personas mayores en Colombia, ajustados según determinantes estructurales e intermediarios del envejecimiento saludable.

**Métodos.:**

Este estudio empleó datos transversales de 23 694 adultos de más de 60 años provenientes de la encuesta nacional Colombia SABE. Se analizaron los determinantes estructurales como las variables demográficas y de situación socioeconómica. Las variables intermediarias se clasificaron en tres bloques: capacidad intrínseca, entorno físico y construido, y sistemas de atención de salud. El análisis de datos empleó la regresión logística con múltiples variables.

**Resultados.:**

La prevalencia de la discapacidad general fue de 21% en la actividad cotidiana, 38% en la actividad cotidiana instrumental y 33% en la discapacidad relacionada con la movilidad. Se asoció la discapacidad con los determinantes estructurales sociodemográficos, como edad avanzada, sexo femenino, residencia en entornos rurales, estar soltero o divorciado, vivir solo, un nivel educativo bajo y tener etnicidad indígena o negra. Con respecto a los determinantes relativos a la situación socioeconómica, la principal influencia en la discapacidad fueron los ingresos netos bajos, la situación socioeconómica baja, una percepción insuficiente de ingresos y un plan de seguro de salud subsidiado. Los determinantes intermediarios de la capacidad intrínseca, como la mala salud informada por la propia persona, la multimorbilidad, la poca fuerza en el puño, un estilo de vida sedentario, las adversidades económicas desde la primera infancia, la carencia de apoyo social y la falta de participación en actividades se asociaron de manera significativa con la discapacidad.

**Conclusiones.:**

Las medidas que tienen un efecto sobre los principales factores asociados con la discapacidad, como la reducción de las inequidades en materia de salud, mediante políticas, estrategias y actividades, pueden contribuir significativamente al bienestar y la calidad de vida de las personas mayores colombianas.

## RESUMO

**Objetivo.:**

Identificar os principais fatores associados à incapacidade em idosos na Colômbia, ajustados de acordo com os determinantes estruturais e intermediários do envelhecimento saudável.

**Métodos.:**

Este estudo usou dados transversais de 23.694 adultos com mais de 60 anos de idade que participaram da pesquisa nacional *SABE Colômbia*. Determinantes estruturais, como variáveis demográficas e de condição socioeconômica, foram analisados. As variáveis intermediárias foram classificadas em três blocos: capacidade intrínseca, ambiente físico e construído, e sistemas de saúde. A análise dos dados empregou regressão logística multivariada.

**Resultados.:**

A prevalência de incapacidade em geral foi de 21% para as atividades da vida diária, 38% para atividades instrumentais da vida diária e 33% para restrição de mobilidade. Constatou-se associação da incapacidade com determinantes estruturais sociodemográficos, como idade avançada, sexo feminino, residência em área rural, estado civil solteiro ou divorciado, viver sozinho, baixa escolaridade e etnia indígena/negra. Com relação aos determinantes da condição socioeconômica, baixa renda líquida, pertencimento ao estrato socioeconômico mais pobre, percepção de renda insuficiente e ter um plano de seguro-saúde subsidiado exerceram grande influência sobre a incapacidade. Determinantes intermediários da capacidade intrínseca, como autopercepção de saúde ruim, multimorbidade, força de preensão reduzida, estilo de vida sedentário, adversidade econômica na primeira infância, ausência de suporte social e não participação em atividades foram significativamente associados à incapacidade.

**Conclusões.:**

Ações que afetem os principais fatores associados à incapacidade, como a redução das iniquidades em saúde por meio de políticas, estratégias e atividades, podem contribuir significativamente para o bem-estar e a qualidade de vida dos idosos colombianos.

Healthy aging is defined as the process of developing and maintaining the functional ability that enables well-being in older age (1). However, aging is significantly associated with the deterioration of an individual’s health and poses a higher risk of disability and mortality (1). Disability is often defined as difficulty or lack of independence in activities of daily living (ADL), such as bathing and dressing, or instrumental activities of daily living (IADL), such as shopping and traveling (2).

Multiple factors, such as demographics, socioeconomic and health conditions, and social and environmental factors, contribute to disability (3). These factors are exacerbated by social determinants of health (SDH); this concept encompasses the full set of social conditions in which people live and work (4). Thus, older people are exposed to inequity and inequality by demographic characteristics, societal factors, and living conditions that determine to some degree the risk of disease, disability, and lower quality of life (5). Therefore, the assessment of SDH has been proposed as an appropriate approach in addressing inequities in health (6). Health inequities refer to the availability of social and health services based on an individual’s personal, financial, and socioeconomic characteristics rather than their health status and needs. Health inequities are defined as differences in health that are unnecessary, avoidable, unfair, and unjust (4).

Colombia is one of the most unequal countries in Latin America, with levels of inequality that are much higher than those in developed countries. In Colombia, the National Census (2018) estimates that approximately 4 million people (9.1% of the population) are 65 years and older. Their health care system is based on a mandatory health insurance model based on managed competition between private insurers. Colombians are covered by one of the two main insurance schemes: the contributory scheme (CS) for formal workers or the subsidized scheme (SS) for those without the ability to pay (7). Recent studies have also shown that inequalities and inequities have grown among Colombian older adults (8–17). Significant associations have been found between indicators of health and SDH, including age and gender (8, 9), skin color (10), chronic conditions (11), frailty (12, 13), functional limitations (14), health care utilization (15), use of psychotropic drugs (16), and social capital (17). All of these factors, together with other SDH, are relevant to understanding the health status of older adults as part of the processes of exclusion and discrimination that have consequences for health inequities in the region.

Multiple measures of population health consequences have also been largely used to compare different populations to identify and quantify health inequalities within and among subgroups (18). Among these health indicators in older adults, the most commonly used are ADL, IADL, and indicators of mobility (18).

The aim of this paper is to identify the main factors associated with disability in older adults in Colombia, adjusted according to structural and intermediary determinants of healthy aging characteristics.

## MATERIALS AND METHODS

The SABE Colombia project (from its acronym in Spanish: *Salud, Bienestar y Envejecimiento*—Health, Well-Being, and Aging) was a cross-sectional study carried out between 2014 and 2015 that involved 23 694 participants aged 60 years or older living in urban and rural communities of Colombia. The details of the SABE Colombia design are described elsewhere (19–21).

The eligibility criteria consisted of men and women (a) who were 60+ years of age, (b) were capable of communicating with the research team, and (c) could provide written informed consent. Individuals were excluded at the beginning of the interview if they had a total score of less than 13 on the revised version of the Folstein Mini-Mental State Examination (MMSE) (22). Low scores on the MMSE were considered indicative of the inability to complete the study procedures, and therefore, a proxy interview was developed. The percentage of interviews applied to proxies was 17.5% (19). The study received ethics approval from the ethics committees, and informed consent was obtained from all participants.

A recent conceptual framework of SDH and healthy aging proposed by Sadana et al. was used to analyze the relationship between variables (23). This approach included the assessment of *structural determinants* of health inequities; the structural determinants included in this study consist of demographic and standard categories of socioeconomic position (SEP) (23). These SDH inequities in turn operate through a set of *intermediary determinants*. The main categories of intermediary determinants of health in this study included intrinsic capacity, physical and built environment, and health care systems (23). Finally, the indicator of predicting *health outcomes* is defined as a decline in function being experienced, which is often measured by NAGI mobility or ADL and IADL disability (18). The three above-mentioned measurements of outcomes were analyzed.

### Independent variables

**Structural determinants.** The sociodemographic variables were age and gender, place of residence (urban and rural), marital status (married vs. single/divorced/widowed), and household structure, including the number of persons living with the respondent (range 0–30), which was dichotomized as either living alone or with at least one other person. Education, which was measured as the highest educational level attained, was grouped into four categories (none or less than primary, primary, less than secondary and secondary, or higher). For ethno-racial identification, we used a question about one’s self-recognition as either Indigenous, Black, Mulatto, Mestizo, or White (12).

In this study, several groups of SEP variables were assessed, including occupational status in the last month (occupied or unoccupied) and monthly family income, which was based on the national minimum monthly salary (MS) value in 2015 (US$ 269) (https://countryeconomy.com/national-minimum-wage/colombia?year=2015); insufficient income is considered a valid measure of current adversity. The self-reported question, “To what extent does your income allow you to meet your needs?” was used, and the possible answers were very well, suitably, not very well, and not at all. Answers were recoded into two categories: sufficient (very well and suitably) and insufficient (not very well and not at all) (9). Socioeconomic status (SES) was determined on a scale ranging from 1 to 6 based on the housing stratum, with 1 representing the highest level of poverty and 6 representing the greatest wealth: 1, the poorest; 2, 3–4, and 5–6, the wealthiest. Health insurance (or social security affiliation) was categorized as either of the abovementioned subsidized or contributory schemes (15).

**Intermediary determinants.** According to the conceptual framework, these variables were classified into three blocks: intrinsic capacity, physical and built environment, and health care systems.

Health status variables included perceived health status, physiobiological markers, chronic conditions, lifestyle, critical events or states during the life course, and social capital. Self-rated health (SRH) was assessed by asking, “How would you evaluate your current health?” (response options were very good, good, fair, bad, or very bad). Physiobiological markers included body mass index (BMI), grip strength, and walking speed. BMI was calculated as weight (kg)/height (m)^2^ and used as a continuous variable that was categorized as follows: underweight (<18.5), normal weight (18.5–24.9), overweight (25.0–29.9), class I obesity (30.0–34.9), class II obesity (35.0–39.9), and extreme or class III obesity (≥40). Grip strength was assessed with a handheld dynamometer, using the sum of the highest values of two measurements on each hand. The lowest quartile stratified by sex and BMI was used as the cutoff to indicate low grip strength. The presence and number of seven chronic conditions (hypertension, diabetes mellitus, heart disease, stroke, chronic obstructive pulmonary disease (COPD), arthritis, and cancer) were ascertained through self-report. Multimorbidity was considered the presence of two or more morbidities. Current physical activity was assessed by an adapted version of Reuben’s Advanced Activities of Daily Living scale (24). Childhood adversity was measured via the following questions on events occurring during the first 15 years of life: early childhood economic adversity was measured with a description of the economic situation of the family and being hungry (25); early childhood social adversity was measured with questions about witnessing or experiencing physical violence in one’s family (25). Regarding social capital, we used two questions reflecting behavioral manifestations of social cohesion or civic participation; for social cohesion, participants were asked about visiting friends and family in the last month (yes/no), and civic participation was estimated by the respondent having attended in the last year (yes/no) a religious association, community center, senior or golden age association, or exercise and physical activity group. These questions have been used in previous studies of social capital (26).

The physical/built environment was evaluated through a subjective built environment and perceived physical neighborhood disorder assessment. The perception (yes/no) about the presence and/or absence of seven infrastructure characteristics for physical and leisure activities was evaluated as follows: uneven sidewalks, parks and walking areas, safe parks, places to sit and rest at bus stops or in parks, public transportation that is close to your home, public transportation for people with disabilities, and adequate disabled people’s parking (27). Perceived physical neighborhood disorder was evaluated with six potential neighborhood problems associated with urban living: crime, lighting at night, traffic, excessive noise, trash and litter, and access to public transportation. Each neighborhood item was counted as a serious problem if the participant responded that the problem was somewhat or very serious. For this analysis, the highest percentage items were considered (28). The use of health services was defined as having at least one medical visit in the previous 30 days and by the occurrence of one or more hospitalizations in the previous 12 months (15).

### Outcome variables

Mobility disability was defined as self-reported difficulty walking 400 m or climbing a flight of stairs without resting (29). ADL was measured using the Barthel Index scale, with 10 basic activities including feeding, bathing, grooming, dressing, fecal and urinary continence, toilet use, transferring, mobility, and use of stairs (30). For IADL assessment, an adapted version recommended for epidemiological studies in elderly people was used (31). This scale included activities such as financial management, housekeeping, shopping for groceries, making telephone calls, taking medication, and using public transportation (31). Disability was defined by reporting difficulties in performing at least one activity.

### Statistical analysis

Study sample characteristics were assessed by using absolute and relative frequencies with 95% confidence intervals (95% CI) for qualitative variables, and measures of central tendency and dispersion were calculated for quantitative variables. Differences in baseline characteristics were compared using independent χ^2^ tests; variables with *p*-values below 20% were included in the adjusted models. A preliminary evaluation of the effect of individual-level variables was performed using a 1-level stepwise logistic model, and significant variables (*p* < 0.05) were included in the multivariate model. The associations and variance between individual variables and ADL, IADL, and mobility disability were evaluated using odds ratios (OR) and 95% CI. For the selection of the variables at the structural and intermediary levels, Wald tests were developed to evaluate their significance regarding disability prevalence.

Finally, multiple logistic regression models adjusted for age and gender were constructed to evaluate the association of structural and intermediary determinants with different levels of disability, including ADL, IADL, and mobility disability. All regression models were estimated with 95% CI and considered the expansion factors of the multistage sampling of the SABE survey. All analyses were carried out using SPSS ™ version 24.0 for Windows.

This study was approved by the Human Ethics Committee of the Faculty of Health of Universidad del Valle (Acts No. 09-014 and 011-015) and the Bioethics Committee of the Universidad de Caldas (code CBCS-021-14).

## RESULTS

**FIGURE 1.**
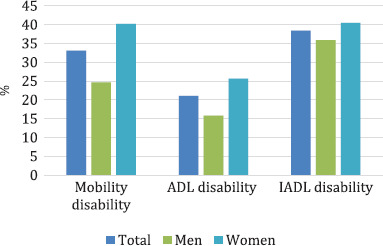
Distribution of outcomes of mobility disability and ADL and IADL functional disability, by gender, SABE Colombia, 2014–2015

ADL, activities of daily living; IADL, instrumental activities of daily living.

***Source:*** Prepared by the authors using SABE Colombia 2014–2015 data.

Table 1 presents the prevalence estimates of mobility, ADL, and IADL disability. The weighted estimates were 33% for mobility, 21% for ADL, and 38% for IADL disabilities (Figure 1). For females, we observed slightly higher prevalence in all domains (mobility, ADL, and IADL). The sample (Table 1) was predominantly female (54.5%). Half of the participants had less than four years of formal education (48.6% of men and 53.4% of women), 90% of the elderly lived with someone, 78% lived in urban areas, 45.9% self-perceived their ethnicity as being Mestizo, 32.5% had been occupied in the previous month, 55.6% of the sample earned less than the minimum salary, and 72.4% considered their income insufficient. Additionally, two out of three persons were very poor (stratum 1–2).

Regarding intrinsic capacity aspects, 57% perceived their health as poor (Table 1). The mean BMI for the total sample was 26.8 ± 5 (overweight); 13% of men and 30.9% of women were classified as obese. The mean grip strength was 20.0 kg (SD 9.7). Multimorbidity was present in 40% of the total sample, and only 21% were regular exercisers. Childhood economic adversity was reported by 27%, while 12% had witnessed or experienced physical violence. Participation in activities reflecting social capital were reported by 50% of the total sample, and 76% had active social network contact in the previous month. The presence of difficulties in the physical/built environment was high, including 90% with perceived built environment problems and 86% reported physical neighborhood disorders. In relation to health care system use, 74% had consulted a physician in the previous month, and 13% had been hospitalized in the last year.

All crude comparisons of individual characteristics showed significant differences among the subjects with and without mobility and disability. The adjusted analysis with structural determinants (Table 2) shows the results of multivariate logistic regression models with outcomes on structural determinants. For mobility disability, factors were related in order as follows: gender (OR = 2.02 [95% CI 2.01, 2.03]), insufficient income (OR = 1.83 [95% CI 1.82, 1.84]), ethnicity (OR = 1.32 [95% CI 1.32, 1.33]), and lower net income, (OR = 1.32 [95% CI 1.31, 1.33]). For ADL disability, the related factors were: insufficient income (OR = 1.72 [95% CI 1.71, 1.74]), lower net income (OR = 1.48 [95% CI 1.46, 1.49]), gender (OR = 1.40 [95% CI 1.39, 1.40]), and ethnicity (OR = 1.40 [95% CI 1.40, 1.41]). Finally, for related IADL disability factors, we found educational level (OR = 2.24 [95% CI 2.20, 2.27]), living alone (OR = 1.90 [95% CI 1.88, 1.91]), ethnicity (OR = 1.63 [95% CI 1.62, 1.63]), and insufficient income (OR = 1.46 [95% CI 1.46, 1.47]) to be related factors.

**TABLE 1.** Distribution of structural and intermediary determinants of inequities, and differences by gender from the SABE Colombia study, 2014–2015

Characteristics	Total *N* = 23 694 (%)	Men *N* = 10 112 (%)	Women *N* = 13 582 (%)	*p*-value
**Sociodemographic factors**				
Age (mean (SD))	69.8 (7.9)	69.5 (7.8)	70.0 (8.1)	<0.0001
Place of residence				
Urban	72.5	67.8	76.1	<0.0001
Rural	27.5	32.2	23.9	
Marital status				
Never married	13.0	9.0	11.3	
Married	53.0	69.8	40.4	<0.0001
Divorced/widowed	35.7	21.0	46.5	
Household structure				
Number of persons living with (mean (SD))	3.1 (2.1)	3.1 (2.0)	3.1 (2.1)	<0.0001
Living alone	9.3	10.8	8.1	<0.0001
Living with someone	90.7	89.2	91.9	
Education				
Education (years) (mean (SD))	4.8 (4.5)	5.0 (4.8)	4.5 (4.3)	<0.0001
Educational level				
None/less than primary	62.4	63.3	62.4	
Primary	16.5	15.7	17.1	<0.0001
Less than secondary/secondary	14.6	14.5	14.6	<0.0001
Higher	6.6	14.5	5.8	
Ethnicity (self-perception)				
Indigenous	6.8	9.4	7.9	
Black	8.2	8.6	8.4	
Mulatto	3.2	3.8	3.5	<0.0001
White	28.8	25.7	27.4	
Mestizo	41.8	44.0	42.8	
Other	5.7	5.2	5.5	
**Socioeconomic position**				
Occupational status				
Occupied in the last month	33.7	51.6	20.4	<0.0001
Net household income				
Lower than 1 minimum monthly salary (MS)	67.5	61.2	72.6	
1 MS	15.9	17.9	14.2	<0.0001
>1–2 MS	9.6	12.2	7.4	
>2–3 MS	2.9	3.9	2.0	
>3–4 MS	1.1	1.8	0.6	
Current income				
Income perception				
Very well	1.4	1.1	1.7	<0.0001
Suitably	25.3	23.1	27.3	
Not very well	54.3	56.3	52.4	
Not at all	18.1	18.5	17.8	
Socioeconomic stratum				
1–2 (poorest)	81.6	84.1	79.8	
3–4	17.6	15.3	19.4	<0.0001
5–6 (wealthiest)	0.7	0.6	0.8	
Health medical insurance				
Contributory	37.4	35.5	38.9	<0.0001
Subsidized	59.8	60.8	59.0	
No insurance	2.2	3.0	1.5	
**Intrinsic capacity**				
Self-rated health				
Good	38.5	43.3	34.9	<0.0001
Regular/poor/very poor	61.5	56.7	65.1	
Physiobiological markers				
BMI (kg/m^2^) (mean (SD))	26.8 (5.0)	25.7 (4.3)	27.8 (5.0)	
Underweight	3.3	3.5	3.2	<0.0001
Normal weight	36.6	44.5	30.3	
Overweight	38.0	38.3	37.8	
Class I obesity	16.8	11.6	20.9	
Class II-III obesity	5.3	2.0	7.8	
Grip strength (kg) (mean (SD))	17.6 (9.2)	22.9 (9.9)	13.9 (6.6)	<0.0001
Medical conditions				
No morbidity	28.4	37.5	21.7	<0.0001
1 morbidity	33.8	33.5	33.9	
Multimorbidity (≥2)	37.8	29.0	44.4	
Lifestyle and behaviors				
Physical activity				
Exercisers (at least 3 times per week)	21.8	28.3	16.4	<0.0001
Frequent long walkers	52.4	64.3	42.4	
No physical activity (sedentary)	25.8	7.4	41.2	
Critical events or states (childhood adversity)				
Early childhood economic adversity or being hungry	26.4	28.3	24.8	<0.0001
Witnessed or experienced physical violence	12.7	12.9	12.5	<0.0001
Social capital				
Participation in activities (yes)	54.6	57.9	51.9	<0.0001
Social networks (yes)	76.0	77.3	75.0	<0.0001
**Physical/built environment**				
Subjective built environment (at least 1 problem)	90.2	89.4	90.9	<0.0001
Safety/security (perception of problems yes)	86.1	85.8	86.4	<0.0001
**Healthcare systems use**				
Medical visit in the previous month (yes)	74.4	74.7	74.1	<0.0001
Hospitalization in the previous 12 months (yes)	13.0	12.7	13.2	<0.0001

BMI, body mass index. Medical conditions: hypertension, diabetes, heart attack, stroke, COPD, arthritis, and cancer. The p-values were obtained by chi-square and t-test.

***Source:*** Prepared by the authors using SABE Colombia 2014–2015 data.

Table 3 shows the results of multivariate logistic regression models for association with disability outcomes on intermediary determinants. For mobility disability, multimorbidity (OR = 3.00 [95% CI 2.99, 3.02]), low grip strength (OR = 2.31 [95% CI 2.30, 2.33]), lack of physical activity (OR = 1.76 [95% CI 1.75, 1.77]), and economic adversity in childhood (OR = 1.71 [95% CI 1.70, 1.72]) were associated. For ADL disability, the associated variables were multimorbidity (OR = 2.82 [95% CI 2.81, 2.83]), hospitalization in the previous year (OR = 2.16 [95% CI 2.15, 2.18]), low grip strength (OR = 2.02 [95% CI 2.00, 1.95]), and no social support or networks (OR = 1.72 [95% CI 1.72, 1.73]). Finally, for IADL, low grip strength (OR = 1.92 [95% CI 1.91, 1.94]), economic adversity in childhood (OR = 1.83 [95% CI 1.83, 1.84]), multimorbidity (OR = 1.69 [95% CI 1.68, 1.70]), and no social support or networks (OR = 1.66 [95% CI 1.66, 1.67]) were the most important factors.

Regarding the physical/built environment, uneven sidewalks or other walking areas were associated with mobility and ADL disability. Perception of neighborhood problems was associated with disability, while lighting at night and excessive noise were associated with mobility, ADL, and IADL disability.

**TABLE 2.** Multivariate logistic regression models for association between structural determinants and mobility disability, ADL and IADL disability, SABE Colombia, 2014–2015

Structural determinants	Mobility disability OR (95% CI)	ADL disability OR (95% CI)	IADL disability OR (95% CI)
**Sociodemographic factors**			
Age	1.10 (1.10, 1.08)	1.10 (1.10, 1.11)	1.10 (1.10, 1.10)
Sex (women)	2.02 (2.01, 2.03)	1.40 (1.39, 1.40)	0.89 (0.89, 0.90)
Place of residence (rural)	1.13 (1.13, 1.14)	1.23 (1.22, 1.23)	1.38 (1.37, 1.39)
Marital status (never married, divorced)	1.03 (1.03, 1.04)	1.36 (1.36, 1.37)	1.31 (1.30, 1.31)
Household structure (living alone)	1.03 (1.03, 1.04)	1.32 (1.31, 1.33)	1.90 (1.88, 1.91)
Educational level (none, less than primary)	1.29 (1.24, 1.28)	1.11 (1.09, 1.13)	2.24 (2.20, 2.27)
Ethnicity (self-perception) (Indigenous/Black)	1.32 (1.32, 1.33)	1.40 (1.40, 1.41)	1.63 (1.62, 1.63)
**Socioeconomic position**			
Occupied in the last month (no)	1.94 (1.81, 2.09)	2.44 (2.20, 2.69)	1.56 (1.46, 1.67)
Income: lower than 1 MS	1.32 (1.31, 1.33)	1.48 (1.46, 1.49)	1.39 (1.38, 1.40)
Poorest socioeconomic stratum (1–2)	1.12 (1.12, 1.13)	1.18 (1.17, 1.18)	1.14 (1.13, 1.14)
Insufficient income	1.83 (1.82, 1.84)	1.72 (1.71, 1.74)	1.46 (1.46, 1.47)
Health medical insurance (subsidized)	1.15 (1.15, 1.15)	1.10 (1.10, 1.11)	1.25 (1.25, 1.26)

ADL, activities of daily living; IADL, instrumental activities of daily living; MS, monthly minimum salary.

Variables in the model: Age, Sex, Place of residence, Marital status, Household structure, Educational level, Ethnicity, Occupied in the last month, Income, Socioeconomic stratum, Sufficient income, Health medical insurance.

***Source:*** Prepared by authors using SABE Colombia 2014–2015 data.

**TABLE 3.** Multivariate logistic regression models for association between intermediary determinants and mobility disability, ADL and IADL disability, SABE Colombia, 2014–2015

Intermediary determinants	Mobility disability OR (95% CI)	ADL disability OR (95% CI)	IADL disability OR (95% CI)
**Intrinsic capacity**			
Self-rated health (regular/poor/very poor)	3.35 (3.13, 3.59)	2.87 (2.64, 3.12)	2.51 (2.36, 2.66)
Obesity (yes)	1.09 (1.08, 1.09)	0.74 (0.73, 0.74)	0.66 (0.66, 0.67)
Low grip strength (<12 kg)	2.31 (2.30, 2.33)	2.02 (2.00, 1.95)	1.92 (1.91, 1.94)
Multimorbidity (≥2)	3.00 (2.99, 3.02)	2.82 (2.81, 2.83)	1.69 (1.68, 1.70)
Lifestyle (lack of physical activity)	1.76 (1.75, 1.77)	1.71 (1.70, 1.73)	1.57 (1.56, 1.58)
Economic adversity or being hungry (yes)	1.71 (1.70, 1.72)	1.63 (1.62, 1.64)	1.83 (1.83, 1.84)
Witnessed or experienced physical violence (yes)	1.12 (1.02, 1.23)	1.28 (1.15, 1.43)	1.51 (1.38, 1.64)
No social activity participation	1.35 (1.35, 1.36)	1.35 (1.34, 1.35)	1.57 (1.57, 1.58)
No social support or networks	1.69 (1.68, 1.70)	1.72 (1.72, 1.73)	1.66 (1.66, 1.67)
**Physical/built environment**			
Subjective built environment (at least one problem)	0.90 (0.82, 1.00)	1.10 (1.00, 1.21)	0.82 (0.76, 0.89)
Safety/security (perception of problems)	1.01 (0.94, 1.10)	1.22 (1.11, 1.34)	1.22 (1.13, 1.31)
**Health care systems use**			
Medical visit in the previous month (yes)	1.22 (1.14, 1.30)	1.18 (1.09, 1.27)	0.84 (0.79, 0.90)
Hospitalization in the previous 12 months (yes)	1.55 (1.54, 1.56)	2.16 (2.15, 2.18)	1.56 (1.55, 1.57)

ADL, activities of daily living; IADL, instrumental activities of daily living.

Variables in the model: Self-rated health, BMI, Grip strength, Physical activity, Childhood adversity (economic and witnessed or experienced physical violence), Social activity participation, Social support, Subjective built environment, Safety/security, Medical visit in the previous month, and Hospitalization in the previous 12 months.

***Source:*** Prepared by the authors using SABE Colombia 2014–2015 data.

## DISCUSSION

The findings of this study reinforce the need for a multidimensional approach to disability studies, in which a myriad of determinants are considered, including disability associated with sociodemographic and SEP structural determinants such as older age, female sex, insufficient income, Indigenous/Black ethnicity, and lower net income. These SDH inequities have been related previously to the accumulated disadvantage, discrimination, and exclusion of older people (23).

Furthermore, a significant association between disability and intermediary determinants was found; these determinants included factors of intrinsic capacity that have previously been related to developing disabilities in elderly individuals, such as multimorbidity, low grip strength, sedentary lifestyle, early childhood economic adversity, hospitalization in the previous year, and no social support or networks (3, 7, 14, 18, 25). Our results reinforce the role of inequities in Latin America; for example, in Brazil, several studies have had similar findings concerning the demographic, socioeconomic, and health factors associated with disability (32, 33).

Older age and being female have been recognized as the most important factors related to developing disability (9). Previously, we hypothesized that mobility and ADL disability differences were related to gender inequalities prevailing in less gender-egalitarian societies (9). Thus, women’s disadvantages in disability could be ascribed to gender inequities in socioeconomic status and multimorbidity (at least half of our sample) in Latin American populations (33). Our results corroborated the important role of ethnicity, Indigenous and Black, in developing mobility and ADL/IADL disability (34). Recently, a study from SABE Colombia showed the social and economic gradients that occur among older adults according to ethnicity; Black or Afro-descendant adults faced the worst social and economic vulnerability, as assessed by their SES and educational level (10). Our results also indicate disability differences between rich and poor populations. Recently, with the same SABE Colombia sample, significant differences in limitation prevalence across national states due to socioeconomic status and demographic characteristics have been demonstrated (14). Furthermore, the subsidized health insurance scheme has been found to be associated with all measures of disability. One study on inequality in health care use among SABE Colombia participants demonstrated that, while there has been progress in extending health insurance coverage for the elderly in Colombia, there are still inequalities in the delivery of health care, especially preventive and outpatient care (15).

Significant associations between disability and several determinants of intrinsic capacity have been found; SRH and multimorbidity are the factors that are the most strongly related to the functional disability of elderly individuals in Colombia. Previously, both factors have also been considered to be the most strongly related to disability among Brazilian elderly individuals (32, 33). Furthermore, in another analysis of SABE Colombia participants, it was possible to identify the existence of a substantial and marked SES gradient in high blood pressure and several additional risk factors for cardiovascular disease among older individuals (11). Hand grip strength is considered one of the most important measures of muscle strength and a key component for diagnosing frailty (12). Our results reinforce that poor muscle strength in old age is associated with decline, especially mobility disability. In a previous study, an association between sociodemographic factors, such as older age, being female, and having insufficient income, was found to be related to a greater risk of incidence of a worse status of frailty (13). It is generally recognized that lifestyle factors are associated with both morbidity and disability (2). In this study, a sedentary lifestyle was one of the most important factors related to disability, thereby reinforcing a previous report of the importance of physical activity in limiting disability (18). Our results suggest possible effects of cumulative exposures since childhood, especially economic hardship and hunger during the first 14 years. It is probable that early childhood economic adversity affects muscle strength, especially in women, predisposing them to frailty and disability in older age (14). The results of this study indicated, as emphasized in other reports, that more economically disadvantaged locations provide less access and few options of services to the local community, few opportunities to perform leisure activities and socialize with friends, and less social interaction and support between individuals and thus contribute to the development of disabilities (27).

Regarding health care system use determinants, our results regarding attending medical visits in the previous month as a protector determinant reinforce the importance of providing health care to avoid disability (15). In this report, elderly people who had been hospitalized over the last 12 months exhibited a greater association with disability. There is strong evidence of a causal relationship between disability onset and hospitalization, because hospitalized elderly individuals often develop disabilities as a consequence of staying in hospital (32).

This study has several strengths. First, it uses a large sample of older adults to study inequities in health. The large national size of the community permits a stratified robust analysis, with most effects pointing in the same direction. Second, we carried out separate analyses of the three outcomes that represent disability, which are important since the loss of independence in these domains tends to occur in different ways as age increases. Third, the identification of factors associated with the disability of older people provides relevant elements for prevention and intervention measures for promoting healthy aging.

On the other hand, the main limitation of this study refers to the cross-sectional design, which prevented causal inferences. Further longitudinal research is needed to determine whether temporal changes in structural determinants have effects on intermediary determinants and in turn have effects on consequences of healthy aging. Another limitation is that, while we made a first attempt to explain inequities in disability through a healthy aging approach in Colombian older adults, other pathways of association should be investigated (4–6). Future research could investigate the mechanisms through which the structural and intermediary determinants affect ability, also taking into account the particularities of the Colombian reality (9). However, to extend beyond multivariate logistic regression analysis, multilevel analysis or structural equation modeling should be used for better comparison of the associations found with disability.

This study provides strong support for the role of structural and intermediary determinants on developing disability in the elderly population of Colombia. Multiple strategies focusing on the reduction in structural inequities should be encouraged, such as education throughout life, the socioeconomic protection of poor rural female children, the permanence of elderly individuals in the labor market, the avoidance of sedentary lifestyles, the promotion of participation in activities, the reinforcement of social support, and interventions that target inequities related to physical and social neighborhoods. All of these strategies should have the unique goal of avoiding or at least delaying the onset of disability.

## Disclaimer.

Authors hold sole responsibility for the views expressed in the manuscript, which may not necessarily reflect the opinion or policy of the *RPSP/PAJPH* and/or PAHO.
